# Effects of egg-mass-dissolving substances on the post-embryonic development of *Chrysomya megacephala* (Fabricius, 1794) and *Chrysomya putoria* (Wiedemann, 1830) (Diptera: Calliphoridae): Implications for maggot therapy

**DOI:** 10.1371/journal.pone.0361039

**Published:** 2026-10-26

**Authors:** Mariana dos Passos Nunes, João Pedro de Oliveira Lima, Jéssica da Silva Costa, Cláudia Soares Santos Lessa, Renato Geraldo da Silva Filho, Jeronimo Alencar, Valéria Magalhães Aguiar

**Affiliations:** 1 Departamento de Microbiologia e Parasitologia, Instituto Biomédico, Centro de Ciências Biológicas e da Saúde, Universidade Federal do Estado do Rio de Janeiro, Rio de Janeiro, RJ, Brasil; 2 Fundação Oswaldo Cruz, Fiocruz, Instituto Oswaldo Cruz, Laboratório de Diptera, Rio de Janeiro, RJ, Brasil; Institut National de la Recherche Agronomique, MOROCCO

## Abstract

Maggot therapy uses necrophagous dipteran larvae to remove necrotic tissue, requiring strict aseptic protocols. The disruption of egg masses is a crucial step to ensure uniform disinfection; however, chemical agents can cause sublethal effects. This study investigated the impact of three disrupting compounds on the post-embryonic development of *Chrysomya megacephala* and *Chrysomya putoria*. Egg masses were subjected for 10 minutes to the following treatments: papain (0.5%), sodium hydroxide (NaOH 1%), potassium hydroxide (KOH 2%), and a control (distilled water). After hatching, 40 larvae per replicate (n = 640 per species) were monitored for viability, stage duration, feeding cessation rate, larval weight (L3), sex ratio, and morphological normality. Data normality was assessed using the Shapiro–Wilk test; comparisons between treatments were performed using nonparametric Kruskal–Wallis tests followed by Dunn’s post hoc tests, while categorical variables were analyzed using chi-square tests and Fisher’s exact test, with a significance level of p < 0.05. The results revealed species-specific responses. In *Chrysomya megacephala*, there was no significant reduction in viability (57% to 70%) or changes in the pupation rate among the groups. However, NaOH significantly reduced larval weight (67.5 mg) compared to the control (69.9 mg), while papain did not differ significantly from the control (68.5 mg). *Chrysomya putoria*, on the other hand, showed greater sensitivity to alkaline compounds; treatments with NaOH and KOH reduced total viability to 68% (compared to 88–91% in the control and papain groups) and delayed the feeding cessation rate. In both species, papain preserved larval weight gain and maintained developmental stability. The total duration of the life cycle (10–11 days) and the morphological normality of adults were not significantly affected by the treatments. It is concluded that *C. megacephala* exhibits greater physiological resilience to chemical stress in early life stages. Among the agents tested, 0.5% papain emerged as the most promising option for maggot therapy protocols, as it combines efficient dissociation with the maintenance of larval biological quality.

## Introduction

The high incidence of chronic wounds and infections caused by multidrug-resistant bacteria generates economic impacts and compromises patients’ quality of life, requiring a reevaluation of current therapies [1–3]. In this context, from the late 1990s to the early 2000s, a biotherapy based on the controlled use of dipteran larvae was reintroduced, standing out for its efficacy and clinical safety [4–6].

Maggot debridement therapy, or simply maggot therapy (MDT), uses decontaminated necrobiontophagous muscoid larvae to remove necrotic tissue from hard-to-heal wounds [7]. Through the secretion of proteolytic enzymes, larvae liquefy devitalized tissue for subsequent ingestion while preserving healthy tissue. This mechanism provides rapid and highly selective debridement, superior to conventional mechanical or surgical approaches [8]. Additionally, larval excretions possess antibacterial and anti-biofilm activity, combating pathogens such as methicillin-resistant *Staphylococcus aureus* (MRSA) and reducing malodor [9]. Together, these actions modulate the wound microenvironment, stimulating granulation tissue formation and accelerating the healing process.

The production of therapeutic larvae requires stable laboratory colonies and rigorous protocols to mitigate contamination risks and ensure reproducibility [4,10]. This process includes the collection and incubation of egg masses, followed by their separation, disinfection, and monitoring of development [11,12]. Each step must ensure viability, synchrony, and larval quality, with the initial handling of eggs being one of the most critical phases for successful production [4,5]. Egg dissociation is essential in maggot therapy protocols, as the natural adhesion of egg masses across different blowfly species hinders effective disinfection for clinical purposes [6,13]; thus, individual egg separation optimizes surface exposure to disinfectants.

Various compounds have been used to dissociate blowfly egg masses, ranging from detergent solutions to specific chemical agents, aiming to facilitate egg handling and disinfection in maggot therapy protocols [4,5,12]. Although practical and efficient, these agents may have limitations when applied at inappropriate concentrations or for inadequate exposure periods [11,12]. Excessive early chemical exposure of embryos can reduce viability, delay development, or cause sublethal effects in later life stages [14,15].

The selection of species for maggot therapy should consider their efficiency in debridement, ease of laboratory rearing, predictability of the life cycle, and tolerance to dissociation and decontamination processes [10,15]. However, comparative studies involving species abundant in tropical regions remain scarce, especially in countries with limited resources allocated to public health. Species of the genus *Chrysomya* Robineau-Desvoidy, 1830, widely distributed in tropical regions worldwide, stand out for their abundance and high synanthropy [16–20]. Despite their recognized potential for use in maggot therapy, data on their response to egg dissociation procedures are scarce, particularly regarding post-embryonic development and subsequent biological quality [21].

This study investigated the effects of three dissociating compounds on the development of *Chrysomya megacephala* (Fabricius, 1794) and *Chrysomya putoria* (Wiedemann, 1830), with a view to their application in maggot therapy.

## Methodology

### Insect collection and colony establishment

To obtain egg masses for the dissociation experiments, laboratory colonies of *Chrysomya megacephala* and *Chrysomya putoria* were established in advance. The colonies were initiated using wild individuals collected at Praça da República (Campo de Santana; 22°54′23″ S, 43°11′19″ W), in the municipality of Rio de Janeiro, Brazil (Fig 1). The insects were captured using entomological traps constructed according to the methodology described by [22]. These were suspended approximately 1.5 m above the ground and left in place for five hours to attract local adult dipterans. No specific legal authorization was required for the collection of the insects, and permission to conduct the collection was obtained from the management of Campo de Santana Park.

**Fig 1:**
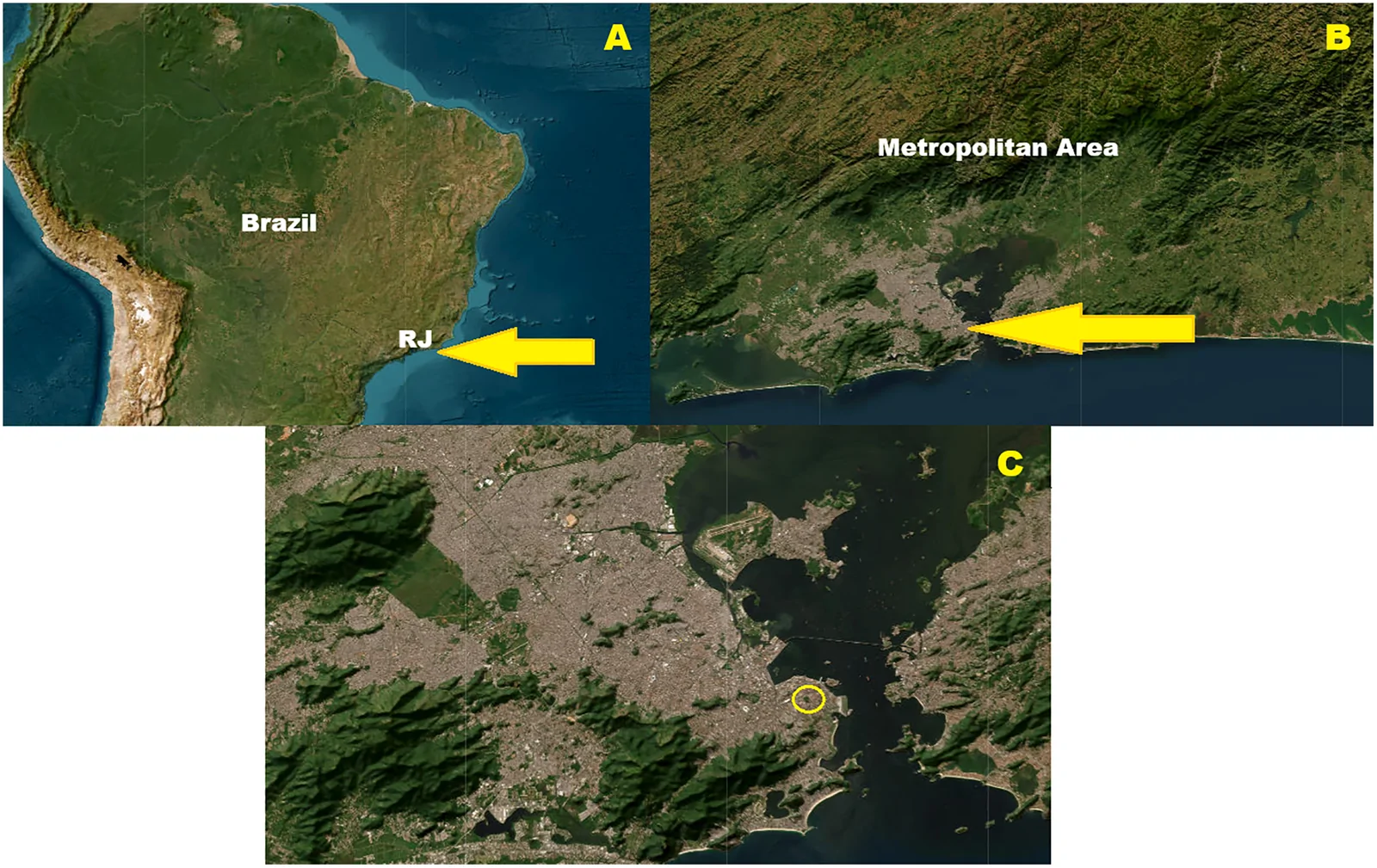
Location of the collection site in the municipality of Rio de Janeiro, RJ, Brazil. (A) Regional map of Brazil, indicating the location of Rio de Janeiro (RJ) on the southeastern coast; (B) closer view of the metropolitan area of the state of Rio de Janeiro; (C) close-up of the state capital, with the collection site at Campo de Santana (22°54′23″ S, 43°11′19″ W) circled in yellow. Satellite images courtesy of the Earth Science and Remote Sensing Unit, NASA Johnson Space Center (https://eol.jsc.nasa.gov/SearchPhotos/).

The captured adults were transported to the Diptera Study Laboratory (LED), affiliated with the Department of Microbiology and Parasitology (DMP) of the Biomedical Institute (IB) at the Federal University of the State of Rio de Janeiro (UNIRIO) (Fig 2), where taxonomic identification was performed according to the key proposed by Kosmann *et al.* [23].

**Fig 2:**
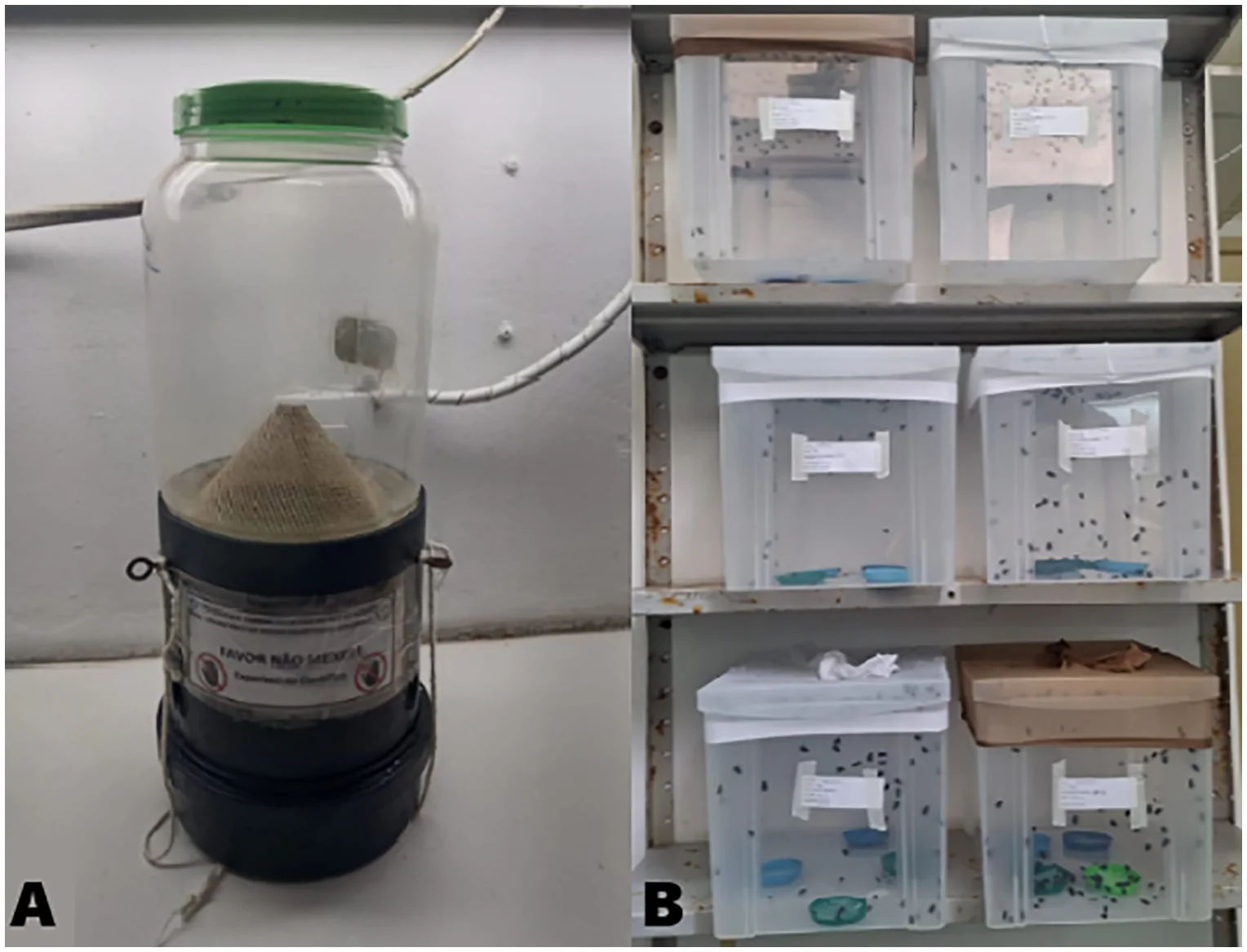
Trap used for collecting Diptera in Campo de Santana, RJ (A); Shelving unit with cages containing adults from the stock colony of *Chrysomya megacephala* at the Diptera Study Laboratory (LED) of UNIRIO (B).

Only identified adults of the selected species were housed in entomological cages measuring 33 × 21.5 × 30 cm (Fig 1B). They were fed with a 50% honey solution and water *ad libitum*, along with a protein-rich diet based on chicken gizzard, used both for ovarian maturation and as a substrate for oviposition. Diets were provided in polyethylene containers (7.5 cm in diameter). Egg masses (0,13–0,18g) laid by females were transferred to 500 mL polyethylene containers containing larval diet (chicken gizzard), at a ratio of approximately 1 g of diet per larva.

### Egg mass preparation and incubation

Individuals of *C. megacephala* (F4) and *C. putoria* (F4) from the stock colonies were used. Egg masses were removed using entomological forceps and transferred to Petri dishes lined with filter paper previously moistened with saline solution (0.9% sodium chloride). The dishes were kept in an incubator under controlled conditions: 21 °C, relative humidity of 70 ± 10%, and a 12 h photoperiod, for approximately 14 hours, corresponding to the egg incubation period.

### Chemical dissociation treatments

To dissociate the egg masses, three chemical substances were evaluated: 0.5% papain solution (Treatment 1), 1% sodium hydroxide (NaOH) (Treatment 2), and 2% potassium hydroxide (KOH) (Treatment 3). The control group consisted of egg masses washed only with distilled water. For each treatment and replicate, a single egg mass weighing between 0.13 and 0.18 g (mean 0.15 g) was used. This weight range was adopted because larger egg masses were difficult to dissociate and handle uniformly, whereas smaller ones required more extensive manual separation, increasing handling time and the risk of damage; additionally, egg masses below 0.13 g risked yielding at or below 160 viable L1 larvae, which would have been insufficient to complete all four replicates of the experiment. Fig 3 illustrates the main steps of egg mass preparation and dissociation.

**Fig 3:**
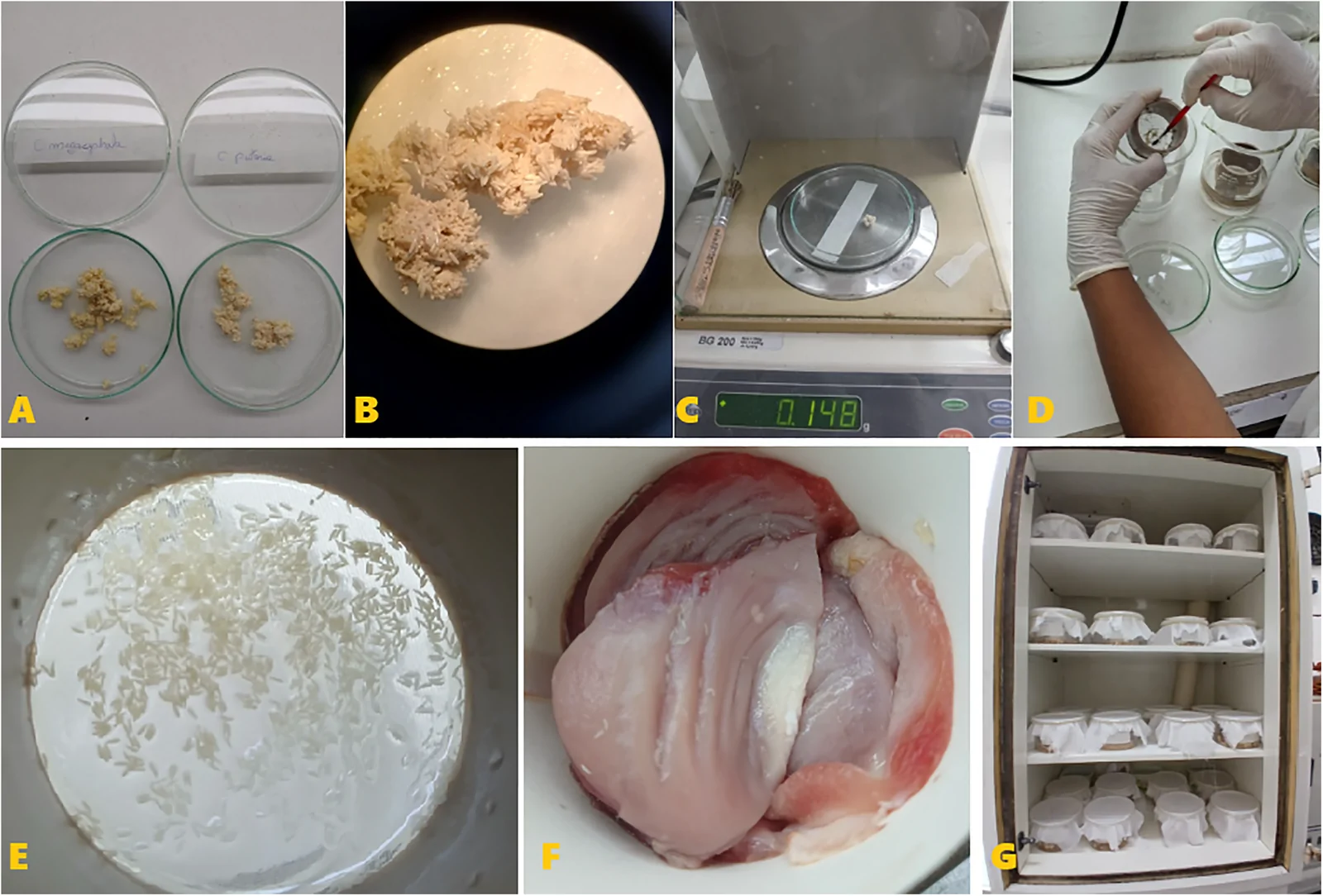
Egg mass of *Chrysomya megacephala* and *Chrysomya putoria* from the stock colony of the Diptera Research Laboratory (LED), as seen with the naked eye (A); egg masses with embryos viewed under a 10x stereomicroscope NIKON SMZ745/ SMZ745T (B); egg masses weighed on a semi-analytical balance (C); dissociation (D); dissociated eggs (E); 40 larvae transferred to a protein diet (F); larval rearing jars kept under a fume hood (G).

For the submersion of egg masses (Fig 1D), handmade sieves were constructed using cylindrical PVC tubes (5 cm in diameter × 5 cm in height), sealed at one end with nylon fabric (escaline) fixed with instant adhesive (Loctite®). The sieves containing egg masses were placed in 200 mL beakers containing the respective dissociating solutions for each treatment, as well as the control. Egg masses remained submerged for 10 minutes. During this period, dissociation was aided using a soft-bristled brush to promote egg separation, combined with circular movements of the sieve performed with entomological forceps. In the control group, egg masses were submerged only in distilled water for the same period.

After exposure to the dissociating agents, egg masses were washed with abundant distilled water using gentle, pressure-free jets applied with a 20 mL syringe. The sieves were then placed on Petri dishes lined with filter paper moistened with saline solution. Approximately four hours later, coinciding with larval hatching, 40 larvae per replicate, with four replicates per treatment and control (n = 640 larvae per species; 1,280 larvae in total across both species), were transferred using a soft-bristled brush to containers with approximately 45 g of chicken gizzard diet.

### Evaluation of biological parameters

After larvae spontaneously abandoned the diet, body mass was measured using a semi-analytical balance in batches of five individuals. Larvae were then transferred to 15 mL test tubes containing sterilized sawdust for pupation. The following biological parameters were evaluated: larval, pupal, and total viability; duration of larval, pupal, and total stages; rate of stage abandonment; body mass of third-instar (L3) larvae; sex ratio; and adult morphological normality.

### Environmental monitoring and statistical analysis

Abiotic variables (temperature and relative humidity) were recorded throughout the experimental period, which was conducted in a fume hood as described by [24], using a datalogger (AKSO AK170).

Microsoft Excel was used for data organization and preliminary analysis, and RStudio version 4.5.2 was used for statistical analyses. The normality of continuous variables was assessed using the Shapiro–Wilk test. As data did not follow a normal distribution, comparisons between treatments were performed using the nonparametric Kruskal–Wallis test, followed by Dunn’s post hoc test. Categorical variables, including sex ratio and adult morphological normality, were analyzed using Fisher’s exact test with Holm–Bonferroni correction. Sex ratio was calculated as SR = F/ (F + M), where F represents females and M males. A significance level of 5% (p < 0.05) was adopted.

## Results

### Environmental conditions

During the experimental period, environmental conditions were continuously monitored using an AKSO AK170 data logger, whose records are presented in Fig 4. The daily mean temperature ranged from 22.0 °C to 25.1 °C, with a mean of 23.3 °C, while relative humidity ranged from 58.6% to 78.7%, with a mean of 75.2% (T = 23.3 °C; RH = 75.2%). Despite these fluctuations over the course of the days, the Spearman correlation analysis of temporal trends did not indicate a statistically significant variation in the average daily temperature over the experimental period (low ρ; p > 0.05). Similarly, the average relative humidity also showed no significant correlation with time (low ρ; p> 0.05).

**Fig 4:**
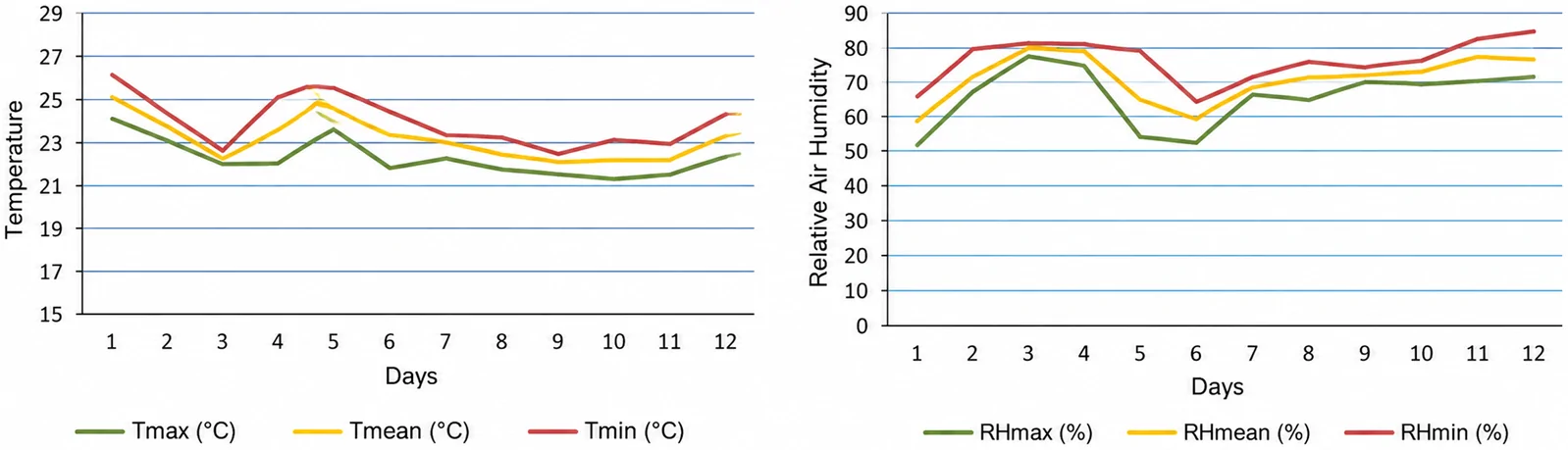
Daily variation in temperature (°C) and relative humidity (%), with minimum, average, and maximum values recorded during the experimental period by an AKSO AK170 data logger.

### Viability

The viability of post-embryonic stages differed between species (Table 1). In *C. megacephala*, no statistically significant differences were observed in larval, pupal, or total viability among treatments (p > 0.05). In all experimental groups, viability remained high, exceeding 70% in the larval and pupal stages and above 57% for total viability. Although numerical variations were observed, with the control treatment showing the highest values and potassium hydroxide the lowest, none of the substances used for egg dissociation caused a statistically significant reduction in viability throughout post-embryonic development.

**Table 1:** Viability (%) of the post-embryonic developmental stages of *Chrysomya megacephala* and *Chrysomya putoria* subjected to different dissociation treatments (T = 23.3 °C; RH = 75.2%).

Species	Treatment	Larval viability (%)	Pupal viability (%)	Total viability (%)
***C. megacephala***	Control	81.25 ± 0.05^a^	81.25 ± 0.05^a^	70.00 ± 0.09^a^
	Papain 0.5%	72.50 ± 0.09^a^	72.50 ± 0.09^a^	63.12 ± 0.10^a^
	NaOH 1%	78.13 ± 0.16^a^	78.12 ± 0.16^a^	66.88 ± 0.12^a^
	KOH 2%	74.38 ± 0.06^a^	74.38 ± 0.06^a^	57.50 ± 0.06^a^
***C. putoria***	Control	97.50 ± 0.02^a^	97.50 ± 0.02^a^	88.75 ± 0.04^a^
	Papain 0.5%	96.88 ± 0.03^a^	96.88 ± 0.03^a^	91.88 ± 0.03^a^
	NaOH 1%	76.88 ± 0.05^b^	76.88 ± 0.05^b^	68.12 ± 0.04^b^
	KOH 2%	75.62 ± 0.05^b^	75.62 ± 0.05^b^	68.12 ± 0.04^b^

**Notes:** Values are presented as mean ± SD (standard deviation). Means within a column followed by the same letter do not differ significantly (p > 0.05; contingency chi-square test followed by Fisher’s exact test with Holm’s correction).

In contrast, *C. putoria* showed statistically significant differences in larval, pupal, and total viability among treatments (p < 0.05). The control and papain groups maintained high viability, exceeding 95% in the larval and pupal stages and above 88% in total viability. Treatments with sodium hydroxide and potassium hydroxide, however, resulted in significant reductions in survival, with total viability around 68%, differing from the control and papain groups, which did not differ from each other.

### Development duration

The duration of post-embryonic development varied depending on the stage analyzed and between species (Table 2). In both species, the larval stage differed significantly among treatments. In *C. megacephala*, treatment with sodium hydroxide (NaOH 1%) resulted in the shortest average larval duration (4.56 days), differing from the control (4.88 days) and papain (4.77 days), while potassium hydroxide (2% KOH) showed an intermediate value (4.68 days) that did not differ significantly from the control. In contrast, in *C. putoria*, treatments with sodium hydroxide and potassium hydroxide significantly increased the larval stage duration, to approximately 4.17 days in both cases, compared to 4.05 days in the control. Papain (4.12 days) was intermediate and did not differ significantly from either group. The total duration of development, from neonate to adult, did not differ among treatments within each species (p > 0.05).

**Table 2:** Duration (days, mean ± SD) of post-embryonic developmental stages of *Chrysomya megacephala* and *Chrysomya putoria* following egg hatching under different chemical treatments (T = 23.3 °C; RH = 75.2%).

Species	Treatment	Larval stage	Pupal stage	Total duration
***C. megacephala***	Control (distilled water)	4.88 ± 0.35^a^	5.13 ± 0.34^a^	11.00 ± 0.00^a^
	T1 – Papain 0.5%	4.77 ± 0.42^ab^	5.22 ± 0.42^ab^	11.00 ± 0.00^a^
	T2 – NaOH 1%	4.56 ± 0.56^c^	5.44 ± 0.56^c^	11.00 ± 0.00^a^
	T3 – KOH 2%	4.68 ± 0.57^bc^	5.32 ± 0.57^bc^	11.00 ± 0.00^a^
***C. putoria***	Control (distilled water)	4.05 ± 0.22^b^	4.95 ± 0.22^a^	10.00 ± 0.00^a^
	T1 – Papain 0.5%	4.12 ± 0.32^ab^	4.88 ± 0.32^ab^	10.00 ± 0.00^a^
	T2 – NaOH 1%	4.17 ± 0.38^a^	4.83 ± 0.38^b^	10.00 ± 0.00^a^
	T3 – KOH 2%	4.17 ± 0.43^a^	4.86 ± 0.35^ab^	10.02 ± 0.16^a^

**Notes:** Values are presented as mean ± SD (standard deviation). Means within a column followed by the same letter do not differ significantly (Kruskal–Wallis test followed by Dunn’s post hoc test with Holm’s correction, p > 0.05).

The prepupal stage was short and biologically stable in both species, lasting approximately 1 day in all treatments and replicates, as consistently recorded during the once-daily monitoring; since observations were made only once per day, sub-day (hour-level) differences between groups, if any, could not have been detected. The total duration of development, from neonate to adult, did not differ among treatments within each species (p > 0.05). However, a consistent interspecific difference was observed, with *C. megacephala* exhibiting a longer average cycle (11 days) compared to *C. putoria* (10 days).

Although the mean duration of developmental stages provides an overall measure of larval time, the rate of diet abandonment allows for an assessment of the temporal dynamics with which larvae reach the L3 stage. In *C. megacephala*, diet abandonment generally began on the fourth day after the start of the experiment, with a peak in occurrence on the fifth day and completion by the sixth day, showing a similar pattern across the evaluated treatments (Fig 5). The treatments with sodium hydroxide and potassium hydroxide showed a higher initial proportion of larvae abandoning the diet on the fourth day; however, the overall temporal pattern converged over the evaluated period. No statistically significant differences were detected in the total rate of larval abandonment among the treatments, indicating temporally stable development up to the L3 stage.

**Fig 5:**
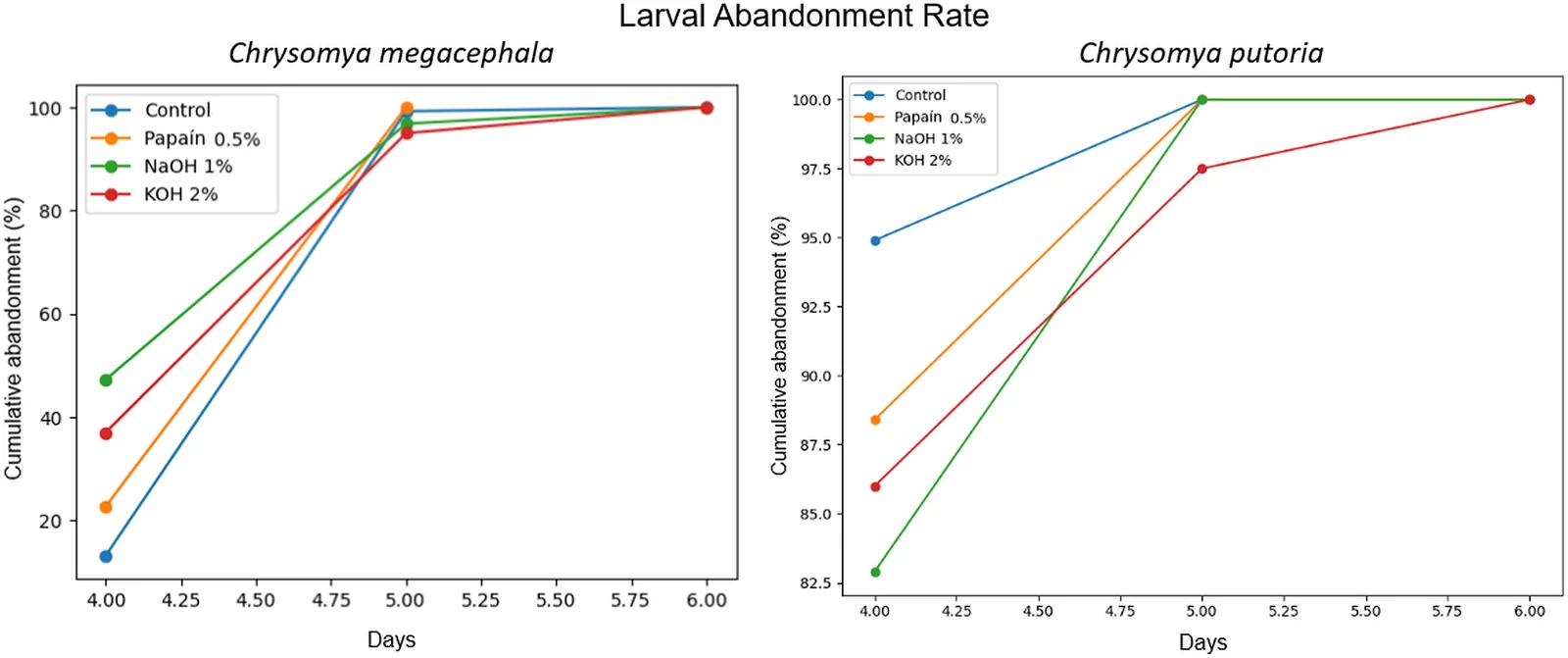
Larval emergence rate (cumulative %) of *Chrysomya megacephala and Chrysomya putoria* over time following egg hatching under different chemical treatments. The curves represent the cumulative proportion of larvae that abandoned the diet (L3 stage) as a function of days of development. Control: distilled water; T1: 0.5% papain; T2: 1% sodium hydroxide; T3: 2% potassium hydroxide.

In contrast, *C. putoria* showed significant variation in the rate of diet abandonment among treatments, with abandonment occurring between the fourth and sixth day after the start of the experiment; however, treatments with sodium hydroxide and, especially, with potassium hydroxide promoted later and temporally more dispersed abandonment compared to the control and papain (Fig 5). This pattern indicates that the alkaline compounds delayed the larvae’s transition to the L3 stage, altering the temporal dynamics of larval development in this species.

The pupation rate was assessed using the cumulative proportion of pupae formed over the days of development. In *C. megacephala*, the Control and Papain groups began pupation predominantly on the fifth day of development, whereas in the hydroxide treatments pupae were already recorded on the fourth day. However, this initial advance did not result in statistically significant differences in the pupation rate among the treatments (Fig 6). The progression to 100% of pupae formed occurred similarly among the groups. Adult emergence was highly synchronized, occurring on the thirteenth day in all treatments, indicating that the compounds used to break egg hatching did not interfere with the total development time to the adult stage.

**Fig 6:**
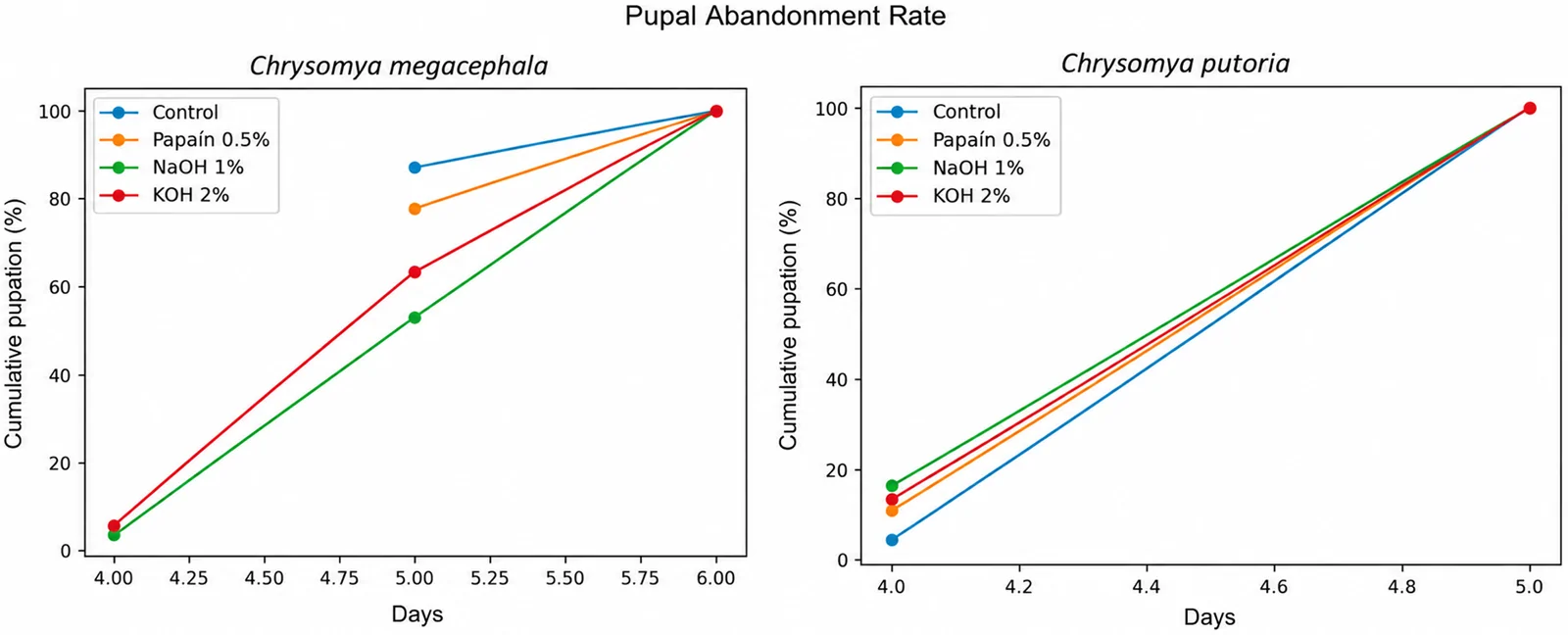
Pupation rate (cumulative %) of *Chrysomya megacephala* and *Chrysomya putoria* over time following egg hatching with different chemical treatments. The curves represent the cumulative proportion of pupae formed as a function of days of development. Control: distilled water; T1: 0.5% papain; T2: 1% sodium hydroxide; T3: 2% potassium hydroxide.

For *C. putoria*, pupation began predominantly on the fourth day of development in all evaluated treatments, with rapid progression to 100% of pupae formed on the fifth day. The cumulative pupation curves showed a highly similar pattern among Control, Papain, and the hydroxide treatments, with no statistically significant differences in the pupation rate detected among the experimental groups (Fig 6). These results indicate that, although the alkaline compounds affected the earlier stages of *C. putoria’s* development, the pupation process remained temporally stable and synchronized.

### Larval weight (L3)

Analysis of the weight of mature larvae (L3) revealed statistically significant differences between treatments in both species (Table 3). In *C. megacephala*, treatment with sodium hydroxide (1% NaOH) resulted in significantly lighter larvae compared to the control, papain, and potassium hydroxide. The other treatments did not differ from one another. The means ranged from approximately 67 mg (NaOH) to 80 mg (papain), indicating that NaOH had a specific negative effect on larval biomass gain, while the control, papain, and KOH showed similar performance.

**Table 3:** Mean weight (mg ± SD) of mature larvae (L3) of *Chrysomya megacephala* and *Chrysomya putoria* after egg hatching with different chemical treatments (T = 23.3 °C; RH = 75.2%).

Treatment	*C. megacephala* (mg ± SD)	*C. putoria* (mg ± SD)
**Control (distilled water)**	69.9 ± 5.2^ab^	60.4 ± 4.6^a^
**Papain 0.5%**	68.5 ± 7.3^a^	60.7 ± 7.5^a^
**NaOH 1%**	67.5 ± 5.5^c^	59.2 ± 2.2^b^
**KOH 2%**	69.7 ± 7.0^b^	58.2 ± 2.3^c^

Notes: Values presented as mean ± SD (standard deviation). Means followed by the same letter in the column do not differ statistically from one another (Kruskal–Wallis test followed by Dunn’s test with Holm’s correction, p > 0.05).

In *C. putoria,* differences were also observed among the treatments. The control and papain treatments had the highest mean weight values (60–66 mg), with no significant difference between them. Treatment with NaOH resulted in a moderate reduction in larval weight (59 mg), while KOH resulted in the lowest mean weight (58 mg), differing significantly from the other treatments. Thus, in *C. putoria*, both alkaline compounds reduced biomass gain, with KOH being the most detrimental.

The boxplot of *C. megacephala* larval weight (Fig 7) illustrates differences in the distribution patterns among treatments. The Control group had a median of approximately 70 mg and a relatively compact distribution. Papain 0.5% showed a similar median, with considerable overlap relative to the Control, explaining the lack of a statistically significant difference. The NaOH 1% treatment displayed a lower median and a distribution shifted toward smaller values, resulting in a significant difference, while KOH 2% showed a slightly higher median and greater dispersion, differing significantly from Papain 0.5% but not from the Control.

**Fig 7:**
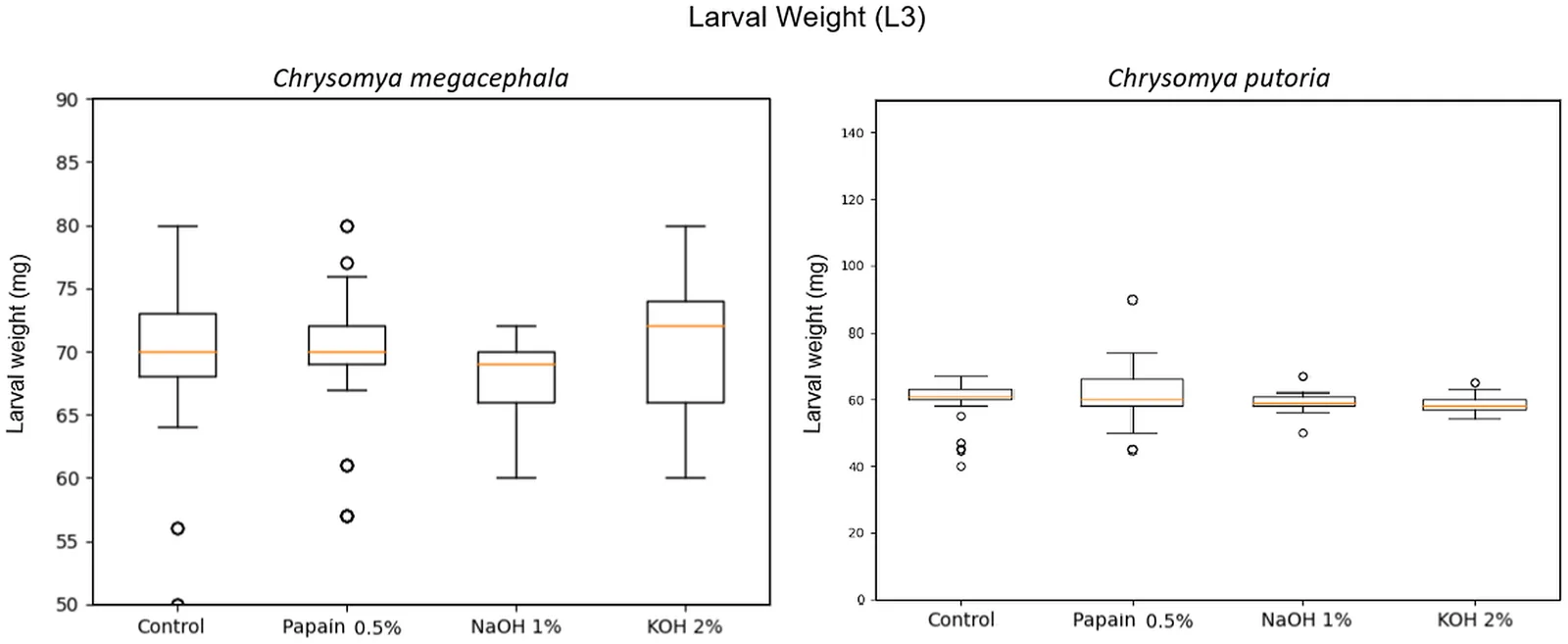
Boxplot of L3 larval weight in milligrams for *Chrysomya megacephala and Chrysomya putoria* after egg hatching with different chemical treatments. The boxes represent the interquartile range, the central line indicates the median, and the points represent extreme values.

In contrast, for *C. putoria* (Fig 7), a clear shift in the distributions was observed in the alkaline treatments. The Control and Papain 0.5% groups presented similar medians (60–65 mg) with broad overlap, showing no statistically significant differences. Conversely, NaOH 1% (59 mg) and KOH 2% (58 mg) displayed lower medians, indicating a systematic reduction in larval biomass. Although partial overlap is present, the medians consistently shifted toward lower values in the hydroxide treatments, reflecting the sublethal effects detected statistically.

### Sex ratio

In *C. megacephala*, the sex ratio remained close to 0.5 across all treatments (Table 4), ranging from 0.464 to 0.485, with no statistically significant differences (p > 0.05). This indicates that the compounds used to dissociate egg masses did not influence the sex ratio in this species. Similarly, in *C. putoria*, the sex ratio also remained relatively stable across treatments, ranging from 0.429 to 0.577, with no statistically significant differences after Holm’s correction (p > 0.05). Although the Control group showed a numerically higher proportion of females (0.577) compared to the treated groups (0.429–0.459), this difference did not reach statistical significance. These results suggest that, within the tested conditions, the compounds used to dissociate egg masses did not significantly influence the sex ratio in either species.

**Table 4:** Sex ratio of emerged adults of *Chrysomya megacephala* and *Chrysomya putoria* following egg-mass dissociation with different chemical treatments (T = 23.3 °C; RH = 75.2%).

Species	Treatment	Females (%)	Males (%)	Sex ratio (F/ [F + M])
***C. megacephala***	Control	46.4	53.6	0.464^a^
	Papain 0.5%	48.5	51.5	0.485^a^
	NaOH 1%	47.7	52.3	0.477^a^
	KOH 2%	47.8	52.2	0.478^a^
***C. putoria***	Control	57.7	42.3	0.577^a^
	Papain 0.5%	42.9	57.1	0.428^b^
	NaOH 1%	45.9	54.1	0.459^a^
	KOH 2%	43.1	56.9	0.431^a^

**Notes:** Letters indicate indicate statistically statistically significant between treatments within each species (chi-square followed by Fisher’s exact test, with Holm’s correction, p < 0.05).

### Morphological normality

Morphological normality was assessed exclusively in emerged adults, classified as normal or abnormal based on the presence of visible external morphological alterations, including wing deformities, body asymmetry, alterations in the cephalic capsule or locomotive appendages, and/or failures in proper cuticle expansion after emergence. In *C. megacephala*, the proportion of normal adults ranged from 85.1% to 93.5% across treatments (Table 5) and remained high in all experimental groups. For *C. putoria*, normality was even higher, with percentages close to or equal to 100% in all treatments, with only a single isolated occurrence of abnormality recorded in the NaOH treatment (99.1%). Fisher’s exact test did not indicate statistically significant differences between treatments in any of the species (p > 0.05). Thus, the small variations observed are consistent with sampling fluctuation, indicating that the compounds used to disaggregate the egg masses did not compromise the morphological development of the adults.

**Table 5:** Morphological normality of adults emerging after egg dissociation with different chemical treatments (T = 23.3 °C; RH = 75.2%).

Species	Treatment	Emerged (n)	Abnormal (%)	Normal (%)
***C. megacephala***	Control	112	11.6	88.4^a^
	Papain 0.5%	101	14.9	85.1^a^
	NaOH 1%	107	7.5	92.5^a^
	KOH 2%	92	6.5	93.5^a^
***C. putoria***	Control	142	0.0	100.0^a^
	Papain 0.5%	147	0.0	100.0^a^
	NaOH 1%	109	0.9	99.1^a^
	KOH 2%	109	0.0	100.0^a^

**Notes:** Values presented as the percentage of normal and abnormal individuals among emerged adults. Means followed by the same letter do not differ statistically from one another within each species (Fisher’s exact test, p > 0.05).

## Discussion

Effective dissociation of egg masses is crucial in maggot therapy to ensure uniform asepsis of the embryonic surface, as highlighted by Dallavecchia et al. [25] and Stadler et al. [26]. In this study, each species responded differently to the chemical treatments. In *Chrysomya megacephala*, variations in total viability (i.e., 57% for KOH, 63% for papain, and 66% for NaOH, compared to 70% in the control)did not result in statistically significant reductions. Larval and pupal viability were similarly unaffected. These findings indicate that all three compounds, applied for 10 minutes, are generally tolerated by *C. megacephala*, allowing effective egg dissociation without detectable lethality. Environmental conditions (temperature and relative humidity) remained stable throughout the experimental period, with no significant temporal trend detected, indicating that the observed treatment effects are not confounded by abiotic variation.

In contrast, *C. putoria* exhibited a significant reduction in total viability under hydroxide treatments (68%), whereas the control and papain groups maintained high viability (88–91%). This pattern confirms that enzymatic agents such as papain are less harmful to the embryonic microenvironment than strong bases, whose aggressiveness may exceed embryonic tolerance and induce cumulative damage during pupation and adult emergence [27,28]. This pattern indicates that, while *C. megacephala* demonstrated greater stability against the tested dissociating agents, *C. putoria* exhibited greater sensitivity to alkaline compounds, reflected in consistent losses throughout post-embryonic development.

The observed interspecific differences suggest greater physiological robustness in *C. megacephala* compared to *C. putoria*, possibly due to variations in chorion thickness, cuticular composition, or detoxification efficiency [29,30]. Among the tested compounds, Although chorion and cuticular composition have not been characterized for these two species, comparative work in other Diptera shows that the degree of chorion/serosal cuticle melanization and thickness can strongly determine an egg’s resistance to external physicochemical stress [31], lending biological plausibility to this hypothesis; direct ultrastructural or biochemical comparison of the egg coverings of *C. megacephala* and *C. putoria* would be needed to confirm it. Interestingly, *C. putoria* naturally breeds in nutrient-rich, ammoniacal substrates such as latrines and decomposing organic waste [32], an environment where some tolerance to alkaline conditions might be expected; its comparatively lower tolerance to NaOH and KOH observed here is therefore not fully explained by its natural breeding ecology and merits further investigation. 0.5% papain emerged as the most promising agent for maggot therapy, combining effective dissociation with minimal biological disruption [33,34].

Previous studies have demonstrated that chemical conditions in the rearing environment can alter survival and development duration. Low concentrations of caustic soda prolonged larval development and reduced adult size in *Lucilia sericata*, while high doses proved lethal, emphasizing the importance of dose, exposure time, and species-specific sensitivity [35]. Although some clinical protocols employ *C. megacephala* [36,37], only a few studies, such as that of Gama *et al*. [37], have assessed the impacts of egg disinfection on subsequent development. This highlights the novelty of evaluating the post-embryonic effects of dissociation treatments.

In *C. megacephala*, despite slight variations in larval stage duration under NaOH treatment (4.56–4.88 days at 23.1 °C), the total development time remained stable, consistent with literature values at similar temperatures [38]. In *C. putoria*, both alkaline treatments delayed the larval stage without affecting the total cycle (~10 days), reflecting the species’ phenotypic plasticity in response to environmental stress [39–42]. Papain maintained normal developmental timing in both species, reinforcing its biological neutrality [39,40]. Although alkaline treatments altered the timing of specific larval stages, total development time was predominantly determined by intrinsic physiological characteristics of each species rather than by the dissociation treatment itself.

The pupal stage was largely unaffected by the treatments, indicating strong physiological stability. In *C. megacephala*, pupal duration (5.1–5.5 days) aligned with previously reported values [38], whereas discrepancies with field studies [43] likely reflect environmental factors such as altitude, humidity, and substrate type. Overall, the stability of pupation and adult emergence rates across treatments suggests robust temporal canalization of development [44–46].

Larval weight analysis revealed that alkaline treatments impaired biomass gain. In *C. megacephala*, only 1% NaOH significantly reduced L3 larval mass, whereas in *C. putoria*, both NaOH and KOH caused reductions, with KOH being the most detrimental. These sublethal effects likely reflect an energy cost associated with chemical stress, diverting resources to detoxification and repair [39,47]. By contrast, papain did not affect larval mass, supporting its practical suitability for maggot therapy [34,48,49]. In comparison, regardless of the treatment, *C. megacephala* exhibited consistently higher larval weights than *C. putoria*, reflecting differences inherent to the body size of the species. However, the response pattern to the treatments differed between them: while in *C. megacephala* the negative effect was concentrated mainly on NaOH, in *C. putoria* both hydroxides had a significant impact, especially KOH.

Sex ratio was unaffected in *C. megacephala*, remaining close to 0.5 in all treatments [47,50]. In *C. putoria*, the Control group showed a numerically higher proportion of females than the treated groups, though the difference was not statistically significant after Holm’s correction, a pattern that would be consistent with differential female sensitivity to early chemical stress [51,52]. A similar bias has been documented in other insects, where females tend to be more sensitive than males to developmental stress, often owing to their typically larger body size and correspondingly higher energy demands during larval growth, which skews surviving cohorts toward males under stressful rearing conditions [53]; in our data, however, this trend did not reach statistical significance (Control vs. Papain, p = 0.014 before correction; not significant after Holm’s correction). Despite this, adult morphology was nearly 100% normal in all groups, demonstrating ontogenetic robustness and compensatory development during metamorphosis [54]. These findings indicate that papain preserves both biological and morphological integrity, whereas hydroxides can induce subtle sublethal effects without causing teratogenic changes.

This study has some limitations. The experiments used the F4 laboratory generation, and colony-rearing conditions differ from those encountered in clinical wounds. Additionally, although sample sizes were adequate for the statistical tests applied, they were limited to a single laboratory cycle, which may present particularities distinct from other cycles. Our findings on developmental duration and larval weight are broadly consistent with previous reports on *Lucilia sericata* exposed to caustic agents [37], reinforcing that dose- and species-dependent sensitivity to alkaline compounds is a general phenomenon among necrophagous Diptera rather than something exclusive to the genus *Chrysomya*. Future studies should evaluate enzymatic activity in larval secretions following chemical exposure (both from dissociating agents, as used in the present study, and from chemical agents specific to egg decontamination) and validate these findings under clinical conditions.

## Conclusion

Chemical dissociation agents affect *Chrysomya* species differently, with *C. putoria* consistently more sensitive to alkaline treatments than *C. megacephala*. Papain 0.5% was the only agent that preserved viability, larval mass, and developmental timing in both species, while sodium and potassium hydroxide caused sublethal effects (particularly reduced viability and larval mass) that were more pronounced in *C. putoria*.

We therefore recommend 0.5% papain over hydroxide-based agents for egg-mass dissociation in *Chrysomya*-based maggot therapy protocols, as it maximizes larval quality without compromising the parameters relevant to therapeutic efficacy. Where hydroxides must be used, species-specific protocols should account for the greater sensitivity of *C. putoria*. Future studies validating these findings under clinical conditions will further support their translation into practice.
